# Toothache and associated factors in Brazilian adults: a cross-sectional population-based study

**DOI:** 10.1186/1472-6831-9-7

**Published:** 2009-02-25

**Authors:** Mirian Kuhnen, Marco A Peres, Anelise V Masiero, Karen G Peres

**Affiliations:** 1 Post-Graduate Program in Public Health, Universidade do Planalto Catarinense, Lages, Brazil; 2 Post-Graduate Program in Public Health, Universidade Federal de Santa Catarina, Florianópolis, Brazil

## Abstract

**Background:**

Toothache is a dental public health problem and one of the predictors of dental attendance and it is strongly associated with the life quality of individuals. In spite of this, there are few population-based epidemiological studies on this theme. Objective: To estimate the prevalence of toothache and associated factors in adults of Lages, Southern Brazil.

**Methods:**

A cross-sectional population-based study was carried out in a sample of 2,022 adults aged 20 to 59 years living in the urban area of a medium sized city in Southern Brazil. A questionnaire including socioeconomic, demographic, smoking, alcohol, and use of dental service variables was applied at adults household. Toothache occurred six months previous of the interview was considered the outcome. Poisson regression analyses were performed following a theoretical hierarchical framework. All analysis was adjusted by the sample design effect.

**Results:**

The response rate was 98.6%. The prevalence of toothache was 18.0% (95% CI 16.0; 20.1). The following variables were associated with toothache after adjustment: female (PR = 1.3 95% CI 1.3; 2.0), black skin colour vs. whites (PR = 1.5 95% CI 1.1, 1.9), low *per capita *income (PR = 1.7 95% CI 1.2, 2.3), smokers (PR = 1.5 95% CI 1.2, 1.9) and those who reported alcohol problems (PR = 1.4 95% CI 1.1; 1.9). To be 40 years of age (PR = 0.5 95% CI 0.4, 0.7) and use dental service in the last year (RR = 0.5 95% CI 0.4, 0.6) were protective factors for toothache.

**Conclusion:**

The prevalence of toothache in adults of Lages can be considered a major problem of dental public health.

## Background

Despite important advances in the oral health indicators of Brazilian children and adolescents, the equivalent epidemiological profile in adults has not changed significantly in the last two decades [1,2]. In adults, the mean of DMF-T index were 22.5 and 20.1 in 1986, and 2003, respectively. Additionally, the Brazilian adults have mainly access to urgent dental services centered on repair or extraction [3].

One of the main consequences of dental problems is toothache. In Brazil (2002–3), toothache was reported by 35.7%, 34.8% and 22.2% of adolescents, adults and the elderly, respectively [2]. Toothache is a dental public health problem and one of the predictors of dental attendance and it is strongly associated with the life quality of individuals [4,5]. This problem can negatively affect the individual's daily activities such as working, having fun and relations with other people [5], to be a cause of sleeping disturb, absence from work and school and refusal of some types of food [6].

Although toothache is a common symptom of dental diseases, significantly affecting individuals and the community [5], few population-based epidemiological studies have been carried out on this theme. An electronic search carried out through the *Medline *database, for the period from 1966 to 2008, using the terms 'toothache', 'dental pain', 'prevalence' and 'epidemiology', showed only two studies [7,8] carried out in Brazil with adults. Eight other were carried out in Canada [5], South Korea [4], Nigeria [9], United States [10-12], China [13], and United Kingdon [14], and Pakistan [15].

This study aims to estimate the prevalence of toothache and associated factors in adults of the urban area of a medium sized city located in the State of Santa Catarina, southern Brazil.

## Methods

A cross-sectional population-based study was carried out in Lages, Santa Catarina State, southern Brazil. Lages is situated in the mountain region, 176.5 km from the state capital, Florianópolis. The city main economic activities are commerce, education and health services. The estimated population for 2006 was 168,382 inhabitants, 97.4% living in the urban area (IBGE – Brazilian Institute of Geography and Statistics).

The population of this study was comprised by all adults aged between 20 and 59 years, of both genders, residents in the urban area of the municipality. The fieldwork was developed from May to October 2007.

In order to calculate the prevalence of toothache the following parameters were used: reference adult population of 86,998, toothache prevalence of 14% [16], a 95% confidence level, and sample error of three percentage points. Since the cluster sample selection was adopted a design effect of 2 was estimated. The calculations included an addition of 10% for compensate non-responses and 20% to control for confounding in multivariable analysis. The minimum required sample size was 1,350 individuals. As this research was nested in a large general health population survey, the final sample size comprised 2,051 individuals. The sampling process was carried out in two stages. The census tracts (n = 186) was the first selection stage and the household as the second. Census tract is the smallest sample unit used in Brazilian censuses each of them presenting approximately the same number of households (300). We randomly selected sixty of them (32.3%). All individuals living in the sampled household were eligible participants of the study. Approximately 17 households were systematically selected within each census tract, resulting in 1,025 households to be visited to achieve the required sample size. Adults living in prisons, nursing homes, hospitals, and those who were unable to answer the questionnaire due to physical or mental reasons were not included in the study. Subjects visited by the research team who could not be found after four visits, including one on weekend and another one in the evening, were considered losses.

The fieldwork was carried out by 10 pairs of interviewers previously trained and blinded to objectives of the study. Master degree students supervised the fieldwork. The data collection was done through a structured and pre-tested questionnaire. A pilot study involving 90 interviews was carried out and an instruction manual about questions and precautions of the field team was developed. The quality control of the data was performed randomly by telephone on 10% of the interviewees.

### Study variables

The dependent variable investigated was the toothache occurring in the six months prior to the interview, obtained through the question: "*In the last 6 months have you had any toothache*?"

The independent variables analyzed were: gender, age, self-reported skin colour, *per capita *income, schooling, self-reported tooth loss, type of dental service and length of time of the last dental attendance, smoking, and alcohol problems.

Age was collected according to completed years of age and categorized in 20–29 years, 30–39 years, 40–49 years and 50–59 years. The self-reported skin colour was categorized as white, dark skinned blacks (*Pretos*), lighter-skinned blacks (*Pardos*), yellow (Asian descent) and Amerindian descent following the criteria adopted by the Brazilian Census.*Per capita income ***was **calculated dividing the family income in Reais (The Brazilian currency) in the month prior to the interview by the number of inhabitants in the household and then transformed into the number of minimum wages (1 minimum wage = R$380 or approximately U$ 224) and categorized according to the frequency distribution quartiles (0.02–0.50, 0.60–0.88, 0.89–1.58, 1.59–19.74). The level of education was collected as a continuous variable (number of school years successfully completed) and divided into four categories (up to 4, 5–8, 9–11 and 12 or more years of study). For the self-reporting of tooth loss the following questions were defined: "*Considering your natural upper and lower teeth, do you have: 10 or more natural teeth, less than 10 teeth or no teeth*?" [17]. Individuals who had been edentulous for more than six months were excluded.

The type of dental service was grouped into public (health center, university and emergency) and private (clinic/private practice). The time since the last dental appointment was analyzed according to the attendance in the year prior to the research. Smoking was categorized into never smoked, current smoker and ex-smoker. Additionally, the number of cigarette packets consumed annually was categorized as less than 10 and 10 or more. The variable "problems with alcohol" was evaluated through the CAGE questionnaire (*Cutting down, Annoyance by criticism, Guilty feeling, and Eye-openers)*, validated in Brazilian Portuguese [18], classifying the individual as "without alcohol problems" (those who responded negatively to all of the CAGE questions) or "with alcohol problems" (those who had at least one positive response to the CAGE).

### Statistical analysis

**Figure 1:**
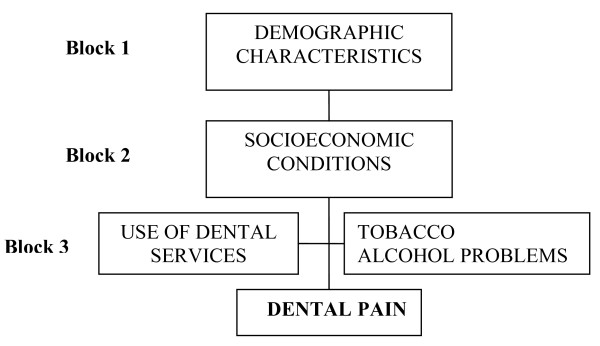
**Hierarchical model to toothache determination**.

The analysis of the factors associated with toothache was carried out considering a theoretical hierarchical model of determination (Figure 1) [19-21]. The demographic characteristics were considered as the most distal factors (Block 1), considering that gender, age and skin colour influence the socioeconomic conditions (Block 2), which in turn determine the use of dental services and the adoption of habits harmful to health, specifically, smoking and alcohol problems (Block 3). Statistical analysis included description of the characteristics of the population, and the variables studied through the frequency distribution, the calculation of prevalence and 95% confidence interval. For the identification of the factors associated with the prevalence of toothache a multivariable analysis was performed using the Poisson regression analysis with binary outcomes allowing the estimation of the prevalence ratios (PR) and their 95% confidence intervals. Poisson regression is recommended in cross sectional study with binary outcome of approximately 20%, so that the odds ratio tends to overestimate the prevalence ratio [22]. All of the analyses considered the effect of the sample design through the *svy *command of STATA 9.0, designed for the analysis of data from complex samples. The variables with p values 0.25 in the bivariate analysis were included in the multivariable analysis and were kept in the model if they remained statistically significant (p < 0.05) or fitted to the model.

This research was approved by the Research Ethics Committee of the Universidade do Planalto Catarinense and the written consent of the participants was obtained.

## Results

The response rate was 98.6% (n = 2,022). Of the interviewees, 187 had lost all of their teeth more than six months prior to the study and, therefore, were excluded from the analysis. Additionally, 30 individuals did not know how to respond regarding toothache, resulting in a final sample of 1,805 individuals.

The average schooling was 9.2 years (standard deviation = 4.2 years) and the average family income was R$1,672.40 or U$ 975 (standard deviation = R$1,657.72). There was a predominance of the female (59.6%); 62.0% of the individuals reported being white, and the average age was 38 years (standard deviation = 11.6 years).

Table 1 shows the sample distribution and prevalence of toothache according to the independent variables investigated. The prevalence of toothache was 18.0% [95% CI 16.0; 20.1]. The highest prevalence of toothache was observed in females (21.4%), among blacks (25.0%) and those who reported to be of Amerindian descent (27.3%). The lower the age, the lower the family income and years of schooling the higher the prevalence of reported toothache. The current smokers showed a higher prevalence of toothache (24.8%) than the ex-smokers (18.5%) and those who had never smoked (14.5%).

**Table 1:** Sample distribution and toothache prevalence according the independent variables (confidence intervals – 95% CI).

**Variables**	**Sample Distribution**		**Toothache**	
	
	**n**	**%**	**Prevalence %**	95 CI % **
**Sex (n = 1,805)**				
Male	728	40.4	13.0	(10.5;15.6)
Female	1,077	59.6	21.4	(18.9;24.0)
**Age group – years (n = 1,802)**				
20 – 29	611	34.0	22.1	(18.0;26.2)
30 – 39	433	24.0	19.9	(16.4;23.3)
40 – 49	458	25.4	13.5	(10.2;16.9)
50 – 59	300	16.6	14.3	(9.3;19.3)
**Skin colour (n = 1,800)**				
White	1,117	62.0	15.9	(13.1;18.7)
Lighter-skinned blacks	515	28.6	20.4	(16.2;24.6)
Dark skinned blacks	112	6.2	25.0	(19.4;30.6)
Yellow	34	2.0	23.5	(9.2;37.8)
Amerindian	22	1.2	27.3	(6.9;47.6)
***Per capita *income-BMW^a ^(n = 1,770)**				
1.59 – 19.74	440	24.8	11.1	(7.7;14.6)
0.89 – 1.58	468	26.4	16.7	(12.3;21.0)
0.60 – 0.88	439	24.8	17.8	(14.4;21.1)
0.02 – 0.50	423	24.0	27.4	(24.0;30.8)
**Educational attainment-years (n = 1,786)**				
≥ 12	445	24.8	12.8	(8.9;16.7)
9 – 11	587	32.9	17.7	(14.3;21.1)
5 – 8	501	28.1	20.1	(16.3;24.0)
≤ 4	253	14.2	22.9	(16.2;29.6)
**CAGE score (n = 1,805)**				
0	1,582	87.6	17.6	(15.6;19.6)
≥ 1	223	12.4	21.1	(15.2;26.9)
**Smoking status (n = 1,802)**				
Never smoked	1,018	56.5	14.5	(12.4;16.6)
Ex-smoked	281	15.5	18.5	(14.2;22.8)
Current smoker	503	28.0	24.8	(20.9;28.8)
**Consumption of cigarettes- Package year (n = 1,805)**				
Never smoked	1,305	72.3	15.4	(13.3;17.5)
<10	100	5.5	30.0	(20.0;40.0)
≥ 10	400	22.2	23.7	(19.4;28.1)
**Number of natural teeth (n = 1,762)**				
≥ 20	1,243	70.4	18.4	(15.9;21.0)
10–19	354	19.9	16.4	(12.8;20.0)
<10	165	9.7	20.3	(14.0;26.0)

**Type of dental service in the last attendance (n = 1,759)**				
Private	1,085	61.0	14.9	(12.4;17.5)
SUS – public	453	27.0	28.7	(24.0;33.4)
Others	221	12.0	12.7	(7.7;17.6)
**Use of dental service in the last year (n = 1,786)**				
Yes	697	39.0	12.3	(10.3;14.4)
No	1089	60.1	21.9	(18.9;25.0)

**Total (n = 1,805)**	1,805	100.0	18.0	(16.0;20.1)

Lages-SC, Brazil, 2007.

* Brazilian Minimum Wage (worth of US$ 200,00).

**confidence interval adjusted for the clustered sampling design.

The prevalence of toothache among the individuals who had used the Unified Health System – SUS (public health service) in the last dental attendance were around twice (28.7%) that of those who had used a private clinic (14.9%).

Table 2 shows the results of unadjusted and adjusted Poisson regression models according to the hierarchical framework (Figure 1). In the unadjusted data analysis, the variables associated with the occurrence of toothache were female, black skin colour compared to white, low *per capita *family income, schooling less than 12 years, smokers, and the use of SUS dental services in the past year compared to those who used private services. Individuals 40 years of age or older and those who attended dental service in the last year were protective factors for toothache.

**Table 2:** Toothache and independent variables.

**Levels**	**Variables**	Model 1* PR (95% CI)	Model 2* PR (95% CI)	Model 3* PR (95% CI)	Model 4* PR (95% CI)
**1**	**Sex**	p < 0.001	p < 0.001		
	Male	1.0	1.0		
	Female	1.6 (1.3;2.0)	1.6 (1.3;2.0)		
	**Age groups-years**	p = 0.004	p = 0.001		
	20 – 29	1.0	1.0		
	30 – 39	0.9 (0.7;1.1)	0.9 (0.7;1.1)		
	40 – 49	0.6 (0.4;0.8)	0.5 (0.4;0.7)		
	50 – 59	0.6 (0.4;0.9)	0.5 (0.3;0.8)		
	**Skin colour**	p = 0.001	p = 0.003		
	White	1.0	1.0		
	Lighter-skinned blacks	1.3 (0.9;1.7)	1.2 (0.9;1.6)		
	Dark skinned blacks	1.5 (1.2;2.0)	1.5 (1.1;1.9)		
	Yellow	1.5 (0.9;2.6)	1.4 (0.7;2.6)		
	Amerindian	1.4 (0.7;3.1)	1.6 (0.7;3.4)		

**2**	***Per capita *income-BMW^a^**	p < 0.001		p = 0.002	
	1,59 – 19,74	1.0		1.0	
	0,89 – 1,58	1.5 (1.0;2.2)		1.3 (0.9;1.9)	
	0,60 – 0,88	1.6 (1.0;2.2)		1.2 (0.9;1.7)	
	0,02 – 0,50	2.4 (1.7;3.4)		1.7 (1.2;2.3)	
	**Educational attainment-years**	p < 0.001		p = 0.083	
	≥ 12	1.0		1.0	
	9 – 11	1.4 (1.1;2.0)		1.2 (0.9;1.7)	
	5 – 8	1.5 (1.7;2.0)		1.4 (0.9;2.0)	
	≤ 4	1.8 (1.2;2.7)		1.6 (0.9;2.7)	

**3**	**CAGE score**	p = 0.156			p = 0.004
	0	1.0			1.0
	≥ 1	1.0 (0.9;1.1)			1.4 (1.1;1.9)
	**Smoking status**	p < 0.001			p < 0.001
	Never smoked	1.0			1.0
	Ex-smoked	1.2 (1.0;1.6)			1.3 (1.0;1.6)
	Current smoker	1.7 (1.4;2.1)			1.5 (1.2;1.9)
	**Consumption of cigarettes- Package year**	p < 0.001			
	Never smoker	1.0			**
	< 10	1.9 (1.4;2.6)			
	≥ 10	1.6 (1.3;2.0)			
	**Type of dental service in the last attendance**	p = 0.049			
	Private	1.0			**
	SUS – public	1.9 (1.5;2.4)			
	Others	0.9 (0.6;1.3)			
	**Use of dental service in the last year**	p < 0.001			p < 0.001
	Yes	1.0			1.0
	No	0.5 (0.4;0.7)			0.5 (0.4;0.6)

^a ^Brazilian Minimum Wage (worth of US$ 200,00).

*Model 1 – crude unadjusted analysis.

*Model 2 – variables in the block 1 adjusted for variables in the same level.

*Model 3 – variables in the block 2 adjusted for variables in the same level and the levels above.

*Model 4 – variables in the block 3 adjusted for variables in the same level and the levels above.

**excluded due lost the statistical significance.

All analysis were adjusted for the clustered sampling design and by the number of teeth.

p value = Wald test.

Poisson regression analysis (Prevalence ratio – PR and confidence intervals- 95% CI). Lages-SC, Brazil, 2007.

After the adjustment of the variables of block 1 (Figure 1) all of the variables remained associated. Individuals of the female gender showed a prevalence of toothache 60% higher than males while blacks present a prevalence of toothache 50% higher than whites. The higher the age the lower the prevalence of the outcome. The *per capita *family income remained statistically and inversely associated with toothache after the adjustment by the variables of blocks 1 and 2. In block 3 the individuals who showed problems with alcohol (PR = 1.4, 95% CI 1.1; 1.9) and the current smokers (PR = 1.5, 95% CI 1.2; 1.9) were associated with the outcome after adjustment by the variables of the previous blocks and the same block. The use of dental services in the past year was a protection factor for the occurrence of toothache (PR = 0.5, 95% CI 0.4; 0.6) even after the adjustment by the distal variables. The variable "number of teeth" was not statistically associated with toothache but was kept in the model since it was a potential confounder.

## Discussion

This study investigated the prevalence of toothache in adults using a representative sample of the adult population of Lages, Santa Catarina State, Southern Brazil. The sample selection criteria, as well as the use of validated data collection questionnaires, the quality control of the data collection, and the unawareness of the interviewers of the objective of this study, reinforces its internal validity. However, the results found in this study can not be infer to other populations due to the socioeconomic contrasts, cultural differences and access to and use of health services found in Brazil.

Cross-sectional studies do not allow measure a causal relationship between the outcome and the independent variables. Another limitation of the study is the possibility of overestimating or underestimating the prevalence of toothache when compared with studies which adopted a history of toothache other than six months. However, other studies have used the same investigation period, which allows the comparison of results [4,16,19]. Additionally, methodological differences such as the age and gender composition of the sample, sample size, along with other studies which included other types of pain, such as orofacial pain, must be taken into consideration on comparing the obtained results.

The prevalence of toothache found in this study was 18.0%, very similar to the prevalence of 17.7% obtained in a population-based study carried out in Pelotas [19], Rio Grande do Sul State, Brazil. A study carried out in the United States of America revealed a prevalence of 14.5% for toothache pain on chewing, in the last six months, in a sample of 28,292 individuals of 20 to 64 years of age [16]. A higher prevalence of 34% was reported in a study carried out in Nigeria, however, a recall period of 12 months was used [9]. Although others studies have been developed using the same way to measure toothache, it is important to consider that other forms of pain from around the mouth, as Temporal Mandibular Disorder can be taking into account.

The results of this study are consistent with those found by other researchers in relation to the association between toothache and the female gender [9,19]. Two hypotheses can be formulated in order to explain this difference. The prevalence of caries and tooth losses, when measured by the DMF-T index, are associated with pain and are more prevalent in women [2]. Women use health services more than men, and are submitted to a greater number of dental interventions and procedures [23], many associated with painful symptomalogies. However, the possibility of temporal ambiguity, inherent in cross-sectional studies, cannot be ignored. The association between gender and toothache is not well established since some studies indicate higher prevalence in males [7], whereas other studies did not find significant differences between genders [4,12,16]. There may be variations in the way in which men and women react to pain; differences related to norms or even biological differences in the mechanisms through which the painful phenomenon is processed [24].

In agreement with other studies, toothache was reported more by young people, decreasing according to age increase [5,16,19]. This is probably because the younger individuals have more teeth and are therefore more prone to be attacked by dental caries and, consequently, have a greater chance of experiencing and reporting pain.

Blacks have shown a prevalence of toothache 50% higher than whites, this value being higher than the 30% identified reported by Bastos *et al *[19]. On the other hand, Riley *et al *[11] did not find difference in the prevalence of toothache between blacks and whites. The Amerindians showed the highest prevalence of pain in this study (60%). These results are examples of the well known and persistent inequalities in health in Brazil. Afro descendants and Amerindian populations tend to show the worst living and health conditions, with less access to adequate food and goods. Hypothetically, the highest prevalence of pain is the result of barriers to access to and use of dental services in these population groups. Research directed toward testing this hypothesis is necessary to better understand the problem.

The prevalence of toothache was associated with low *per capita *family income, consistent with other Brazilian studies which showed the association of dental problems with economic disadvantages [3,7]. Individuals with higher income probably have greater access to measures of preventative health, as fluoridated toothpaste, have better eating and living conditions, acquire more oral hygiene products and, consequently, are less likely to suffer from toothache. Although the schooling variable lost its statistical significance in the adjusted model, it has been the socioeconomic variable most reported in studies relating to toothache [3,9,16,19].

Visiting a dentist in the past 12 months was the protection factor for toothache, in agreement with other studies [7,25]. Hypothetically, visiting a dentist for a routine check up decreases the changes of having pain, implying that routine visits to the dentist may avoid, through different types of treatment that the caries progress to the stage which causes pain [7].

A high percentage (21.9%) of people who reported toothache had not visited a dentist in the past 12 months, suggesting that these individuals bore the pain without resorting to dental services or found other ways to alleviate the pain, such as using analgesics and other medicines. In a study by Vargas *et al *[16], 29.8% of the adults who reported toothache had not visited a dentist in the past year. This highlights, also, that the seeking of services varied significantly according to the socioeconomic characteristics of the population. Toothache is considered to be the best predictor for the seeking of dental services and of the perception for the need for dental treatment [26]. Consequently, the seeking of health services is the most common behavior in response to toothache [5]. Among Brazilians toothache was reported by around 34% of adolescents and 46% of adults and the elderly, as the reason for visiting a dentist [2]. In this study the seeking of a dentist can be in response to toothache or the seeking of whatever dental treatment. Studies with longitudinal outlining could contribute to a better clarify of this relation.

In this study smoking increased the chances of experiencing toothache compared to those who had never smoked. Bastos *et al *[19] found individuals who smoked 20 or more cigarettes per day had 70% more reported pain when compared to non smokers. Riley *et al *[27], in a follow up study, analyzed the associations between smoking and orofacial pain, indicating that smokers showed an increased risk of experiencing painful symptoms. However, after cutting smoking, the risk association with pain decreased significantly, with a decrease in oral disease [27]. The association between smoking and dental caries, the main cause of toothache, has been investigated. Smokers tend to show more accumulation of dental plaque, the saliva may be modified by tobacco, altering its structure which protects teeth during the remineralization of the dental enamel [28]. Root caries seem to be more associated with smoking and there is robust evidence indicating loss of periodontal insertion and, consequently, root exposure to the oral cavity in smokers [28].

The association observed between toothache and problems with alcohol is consistent with other studies [19], however, this must be analyzed carefully. Alcohol consumption may have occurred after the toothache episode in an attempt to relieve it.

## Conclusion

The prevalence of toothache found in adults in Lages may be considered a relevant dental public health problem. Epidemiological studies on toothache may contribute to the improvement of the organization of the health system, the distribution of resources required for oral health promotion and assistance and also the bettering of the education of health professionals. The results of the study indicate a need for structuring of the urgent dental services accessible to the population groups most affected by toothache.

## Competing interests

The authors declare that they have no competing interests.

## Authors' contributions

MK participated in the collection, analysis and interpretation of data, and drafted the manuscript. MAP contributed to conception and design of the study, analysis and interpretation of data, and revising critically the manuscript. AVM helped to draft the manuscript. KGP conceived of the study, performed the statistical analysis, and helped to draft the manuscript. All authors read and approved the final version of the manuscript.

## Pre-publication history

The pre-publication history for this paper can be accessed here:


